# How Clinician-Scientists Access and Mobilise Social Capital and Thus Contribute to the Professional Development of Their Colleagues in Their Networks

**DOI:** 10.1080/28338073.2024.2421129

**Published:** 2024-11-03

**Authors:** Esther de Groot, Jasperina Brouwer, Yvette Baggen, Nienke Moolenaar, Manon Kluijtmans, Roger Damoiseaux

**Affiliations:** a Department of General Practice, Julius Center for Health Sciences and Primary Care, University Medical Center Utrecht, Utrecht, The Netherlands; b Faculty Behavioural and Social Sciences, Department Educational Sciences, University of Groningen, Groningen, The Netherlands; c Education and Learning Sciences Group, Wageningen University & Research, The Netherlands; d Ministry of Education, Culture and Science, Dutch Inspectorate of Education, The Netherlands; e Center for Education, University Medical Center Utrecht, Utrecht University, Utrecht, The Netherlands

**Keywords:** Professional networks, social capital, knowledge exchange, continuing professional development

## Abstract

Clinician-scientists, physicians who conduct research, may fulfil a bridging role in networks of health care researchers and practitioners. Within clinician-scientists’ networks, knowledge sharing is thought to play a vital role in the continuing professional development of themselves and their colleagues. However, little is known about networks of clinician-scientists and how this impacts continuing professional development. Rooted in social capital theory, this study provides a mixed methods exploration of clinician-scientists’ networks. Ego-level social network data were collected via semi-structured interviews on professional interactions about evidence-based practice with 15 clinician-scientists in the area of general practice and elderly care. Quantitative analysis revealed that professional networks of clinician-scientists varied in size, composition, and frequency of interactions depending on appointed research time and experience. Less experienced clinician-scientists interacted most frequently with other clinician-scientists while experienced clinician-scientist experienced more sporadically with clinicians. Clinician-scientists with more research time interacted more frequently with scientists and had a slightly larger professional network than those with less research time. The thematic qualitative analysis revealed different decision-making processes of clinician-scientists on mobilising their social capital and connecting to others in the network: (1) deliberate decision about initiating connections; (2) reactive behaviour without a decision; (3) ad-hoc decision. Clinician-scientists exchange knowledge to enhance their own continuing professional development mainly but also contribute to the professional development of clinicians, scientists, and other clinician-scientists.

## Introduction

Clinician-scientists are practicing clinicians engaged in scientific research [1]. They facilitate knowledge exchange in and between the fields of clinical research and routine clinical practice. On the one hand, they transfer knowledge from the research world towards clinical practice and translate research findings into meaning for practitioners. On the other hand, they share clinical insights and clinically relevant research questions with the research context. Because of their explicit role in clinical practice and scientific research, clinician-scientists are adherents of two professions, dual- or hybrid-role professionals who are increasingly important [2,3].

Clinician-scientists exchange knowledge between clinical practice and research thus impacting the continuing professional development of scientists, clinicians and other clinician-scientists [2,4,5]. Likewise, interactions with these professionals, captured in the clinician-scientists’ social networks, are essential for their own professional development [6]. Previous research has shown that the social networks of clinical teachers benefitted their own professional development [7] and that of their colleagues [8]. The relationships and resources in clinician-scientists’ networks are assumed to be highly relevant for exchanging knowledge between research and clinical practice [9].

Social networks consist of relationships with others that provide access to resources such as skills, knowledge, or social support to deal with challenging circumstances [10–13]. In line with social capital theory [13,14], clinician-scientists may mobilise these resources in the networks. By doing so, they can reach their specific goals and contribute to their own professional development and that of their colleagues. Thus far, the research field provides little understanding of clinician-scientists’ position within a network of other clinicians, scientists, and clinician-scientists. Also, it is unclear how and for which professional development goals clinician-scientists mobilise their social networks [15].

## Social Capital, Professional Networks and Professional Development

Social capital encompasses interpersonal relationships between actors in a network [16]. Loosely defined, social capital refers to the relational resources within a network that are used for different individual purposes (e.g. better performance). Social capital can be distinguished as access to social capital (i.e. the availability of valuable resources in the network) and mobilisation (i.e. making use of resources) [13,14]. It is assumed that individuals with more access to different resources are more likely to share their resources within their networks [13].

Three dimensions of social capital are highly relevant: network size, network composition, and frequency of interaction. The dimensions of “professional-network size” and “professional network composition” are indicators for access to social capital. In contrast, the dimension frequency of interaction in the network indicates the mobilisation of social capital [13]. First, network size reflects the number of professionals with whom a clinician-scientist may interact to exchange knowledge which might be disseminated elsewhere. Research suggests that a more extensive network provides, through the relations, more opportunities for knowledge exchange and better access to resources captured by these relationships [11,13]. Second, the network composition relates to different types of network interactions with clinicians, scientists, and clinician-scientists. A homophily effect whereby people are attracted to connect when individuals are (perceived to be) similar [17]. Homophily is expected to lead to more information sharing. Interpersonal relationships are often multidimensional; for example, collaborating people may develop friendships and meet outside the workplace [18]. Borgatti and Cross (2003) suggested that asking for help or information depends on expertise and convenience in terms of low costs [19]. It seems convenient to ask somebody for advice with whom you have a relationship. Third, the frequency of interactions reflects how often the clinician-scientist interacts with other clinicians, scientists and clinician-scientists. Interestingly, sporadic interactions may be as meaningful as frequent ones – although both contain different values. Sporadic interactions can be considered weak ties, which may provide new information and insights [20].

In addition to differences in network size and composition, exploring individual characteristics related to clinician-scientists’ networks is relevant (i.e. the so-called actor attributes) [21]. For example, access to social capital may depend on work experience. Noben et al. (2022) showed that teachers with more years of work experience collaborated more often within their professional network [22]. Similarly, clinician-scientists with more work experience would have had more opportunities to establish a professional network than clinician-scientist with less experience. Having access to social capital (i.e. more connections with other professionals) has consequences for continuing professional development. For example, clinician-scientists can act as role models for other clinician-scientists. Professional development and learning occur as social processes when employees discuss, reflect, or exchange knowledge and experiences [23–25]. Additionally, the size and composition of clinician-scientists’ networks may depend on the available research time for the clinician-scientist. In research, collaboration with other scientists is paramount [26], but it takes time to develop and maintain these relationships and to have the opportunity to meet clinicians, scientists, and clinician-scientists.

## The Current Study

Using a mixed-method approach, we explored clinician-scientists’ networks to understand their opportunities for knowledge exchange. With such exchange, they have the opportunity to bridge clinical practice and research. Having a dual role potentially brings new opportunities for continuing professional development – especially because of their interactions with colleagues across organisations. This study aims to provide insights into the value of these interactions for the professional development of clinicians, clinician-scientists, and scientists from a social network perspective. We address the following research questions: (1) What are the differences in social capital mobilisation between clinician-scientists with different years since PhD (experience) and percentages of research time in the current position? (2) How do clinician-scientists mobilise social capital: the resources accessible through their professional and social network composed of clinicians, clinician-scientists, and scientists? (3) For what professional development goals do clinician-scientists mobilise social capital in their networks?

## Materials and Methods

We used a mixed-methods design as part of a broader study about clinician-scientists within general practice and elderly care medicine [2,9,27]. Quantitative and qualitative data were collected through semi-structured, in-depth, face-to-face interviews that lasted between 45 and 75 minutes, using a interview guide (see Appendix) [9,27]. During the interview, demographic data, and data on network composition and the frequency of interactions were collected. In addition participants’ ideas about being a clinician-scientist were discussed. The prevailing ethical procedures were followed in line with the NVMO (Dutch Association for Medical Education) which are equal to the principles outlined in the Declaration of Helsinki. For the qualitative part of the research, the Standards for Reporting Qualitative Research (SRQR) were applied [28]. The participants signed a declaration of consent before participating. In the fall of 2017, we conducted interviews with 17 clinician-scientists who were selected using convenience sampling from the researchers’ professional networks which covered many of the clinician-scientists in this domain. Two respondents were left out of the analysis because the data collection of their networks was incomplete, resulting in a total sample of 15 clinician-scientists. Demographics are shown in Table 1. The network visualisations were created using an R script that is available on request.

**Table 1.** Demographics of the participants.

	N
**Sex** Female Male	5 10
**Age** 30-39 40-49 50-59 60-69	3 5 4 3
**Professional background** General practitioner Geriatric physician	9 6
**Years since PhD (Experience)** < 8 ≥ 8	7 8
**Percentage of research time** < 40% ≥ 40%	6 9

To delineate their perceived networks, we asked each participant the following name-generating question: “In the past year, with whom did you exchange knowledge on scientific evidence for clinical practice?” Participants were asked to write down the (first) names on a sticky note. Follow-up questions were posed to elicit as many professional interactions as possible. For each contact, the participant and the researcher identified whether the involved professional was a clinician, scientist, or clinician-scientist and whether someone was considered a friend. Then, the participant was invited to post these sticky notes on a map with three concentric circles, reflecting interactions that respondents perceived to occur frequently (inner circle), occasionally (second circle), or sporadically (outer circle). The first circle was an indication for strong ties, whereas the second and third cycle were an indication for weaker ties.

The quantitative data were analysed descriptively in SPSS (means, SD). Network size of the clinician-scientists’ professional networks was based on the total number of professional relationships that interviewees indicated (i.e. sum scores). Network composition was based on the total number of clinicians, scientists, and clinician-scientists in their professional networks. The frequency of interaction was based on the total number of clinicians, scientists, and clinician-scientists in the inner circle (frequently), the second circle (occasionally), and the outer circle (sporadically) of the network map. Both means and range of these scales, and whether people in the network were similar (clinician-scientist) or not similar (scientist or clinician), were calculated. The qualitative data from the interviews and the ego networks were analysed by the lead researcher (EdG) with the lens of professional development. Other researchers (JB and YB) discussed and agreed upon the themes. Note: the interviews were analysed earlier with another theoretical lens to explore how identity shaped brokerage [27]. For the current study, a qualitative thematic analysis was conducted to explore how and for what professional development goals the clinician-scientists interacted with other professionals within their network. The whole research team maintained reflexivity throughout the analysis and writing process. The quantitative and qualitative data were analysed in an integrated manner. With a cross-case analysis, using the data analysis program NVivo (12 Plus), we explored whether clinician-scientists who differ in their professional network size, years since PhD (< or ≥ eight years), and percentage of research time in their current position (< or ≥ 40%) have different perceptions of and approaches to professional development. We created equal groups in terms of working experience to compare to what extent the network differs for clinician-scientist with less or more experience since they obtained their PhD. In Europe, including the Netherlands, you are considered as an early career researcher or professional when you have less than 8 years of experience.

## Results

### Access and Mobilisation in the Network

Regarding the composition of the clinician-scientists’ professional networks (see Table 2), participants exchanged knowledge mostly with other clinician-scientists and clinicians in their professional networks. On average, they had fewer scientists in their professional network than clinician-scientists and clinicians.

**Table 2.** Descriptive statistics of the size and the composition of clinician-scientists’ professional networks.

Network size	Mean	Min	Max
**Professional network**			
Clinicians	5	0	10
Scientists	3	0	9
Clinician-scientists	6	1	12

*N* = 15 participants (clinician-scientists).

Clinician-scientists’ professional network size varied substantially, with an average of 14 relationships (range = 24), see Table 2. In terms of frequency of interaction, see Table 3, on average, clinician-scientists sporadically interacted slightly more with clinicians (Mean (=M) = 2) With scientists, they interact frequently, occasionally, or sporadically with relatively equal averages (M_scientists_ = 1, see Table 3). In their professional network, frequent interactions occur with other clinician-scientists (M_clinician-scientists_ = 3, vs M_scientists_ and M_clinicians_ = 1).

**Table 3.** Frequency of professional interactions with clinicians, scientists, and clinician-scientists.

Frequency of interactions	Mean	Min	Max	
**Frequently**				
Clinicians	1	0		4
Scientists	1	0		6
Clinician-scientists	3	0		8
**Occasionally**				
Clinicians	1	0		4
Scientists	1	0		3
Clinician-scientists	2	0		5
**Sporadically**				
Clinicians	2	0		8
Scientists	1	0		3
Clinician-scientists	1	0		5

### Experience

Seven clinician-scientists had obtained their PhD less or equal to eight years ago and were considered less experienced than the eight clinician-scientists who obtained their PhD more than eight years ago. The descriptive statistics suggest that the professional network size of experienced clinician-scientists did not differ from less experienced clinician-scientist. In both groups, the clinician-scientists most often interacted with other clinician-scientists (see Table S1 in the Appendices). The means in Table S1 suggests that less-experienced clinician-scientists have nominated slightly more clinician-scientists with whom they interact frequently than more-experienced clinician-scientists. M_more experienced_ = 1; M_less experienced_ = 3. However, more and less experienced clinician-scientists are similar in their number of other clinician-scientists or scientists with whom they sporadically interact, i.e. only one on average, although the distribution is larger for more experienced clinicians. Also, more experienced clinician-scientists nominated a larger number of clinicians, on average, with whom they they sporadically interact, M_more experienced_ = 3; M_less experienced_ = 1. The more experienced clinician-scientists interacted with a larger whole group of people, yet more often on a sporadic basis (see Figures 1a,b).

**Figure 1a.**
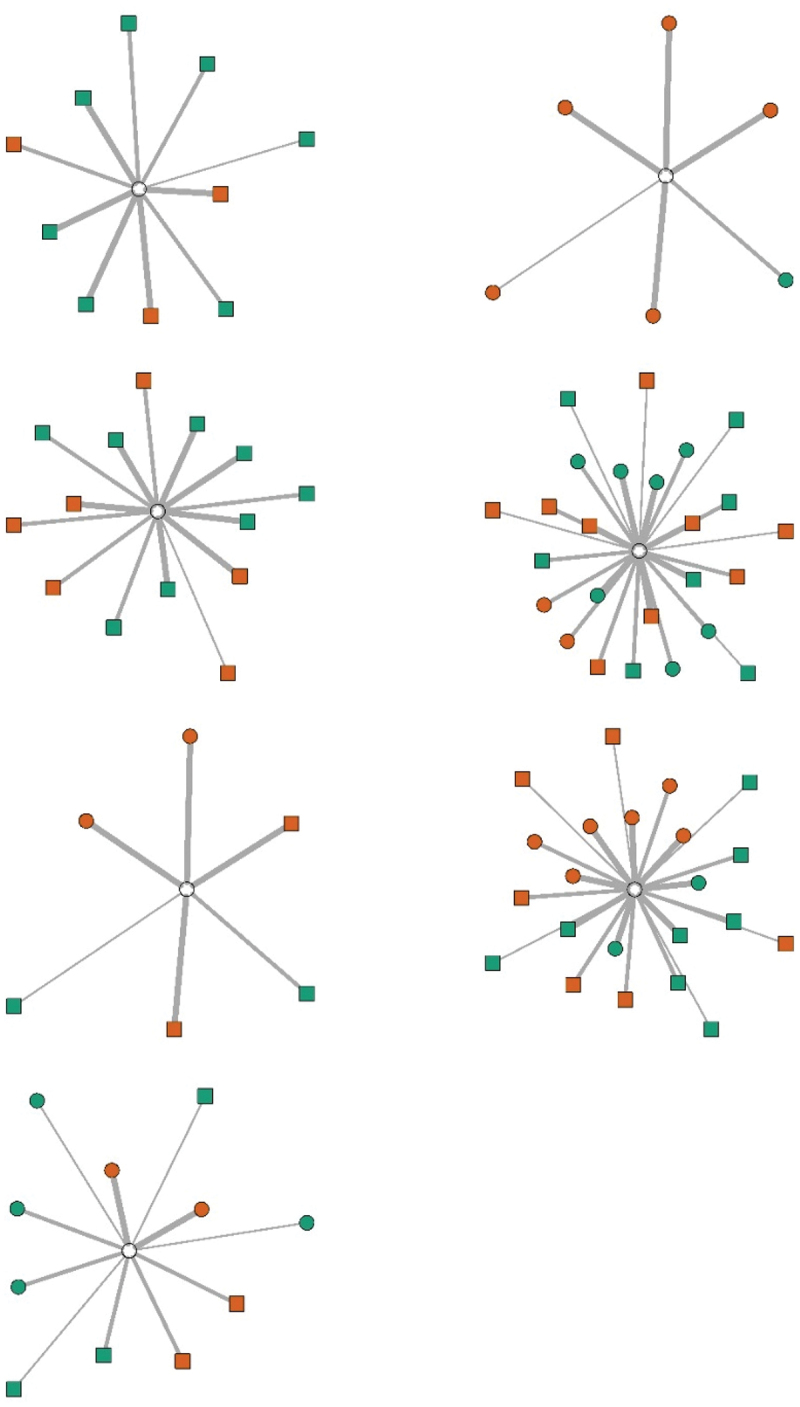
Network visualisations for participants with less than 8 years since PhD. The orange nodes are clinician-scientist which equals same profession while the green nodes are clinician or scientist. The nodes depicted as a circle ware considered friend, and square were not. The thick lines give an impression of frequent, the thin lines of occasional, and the very thin line of sporadical interactions. Data on friendship relations was omitted from the manuscript overall.

**Figure 1b.**
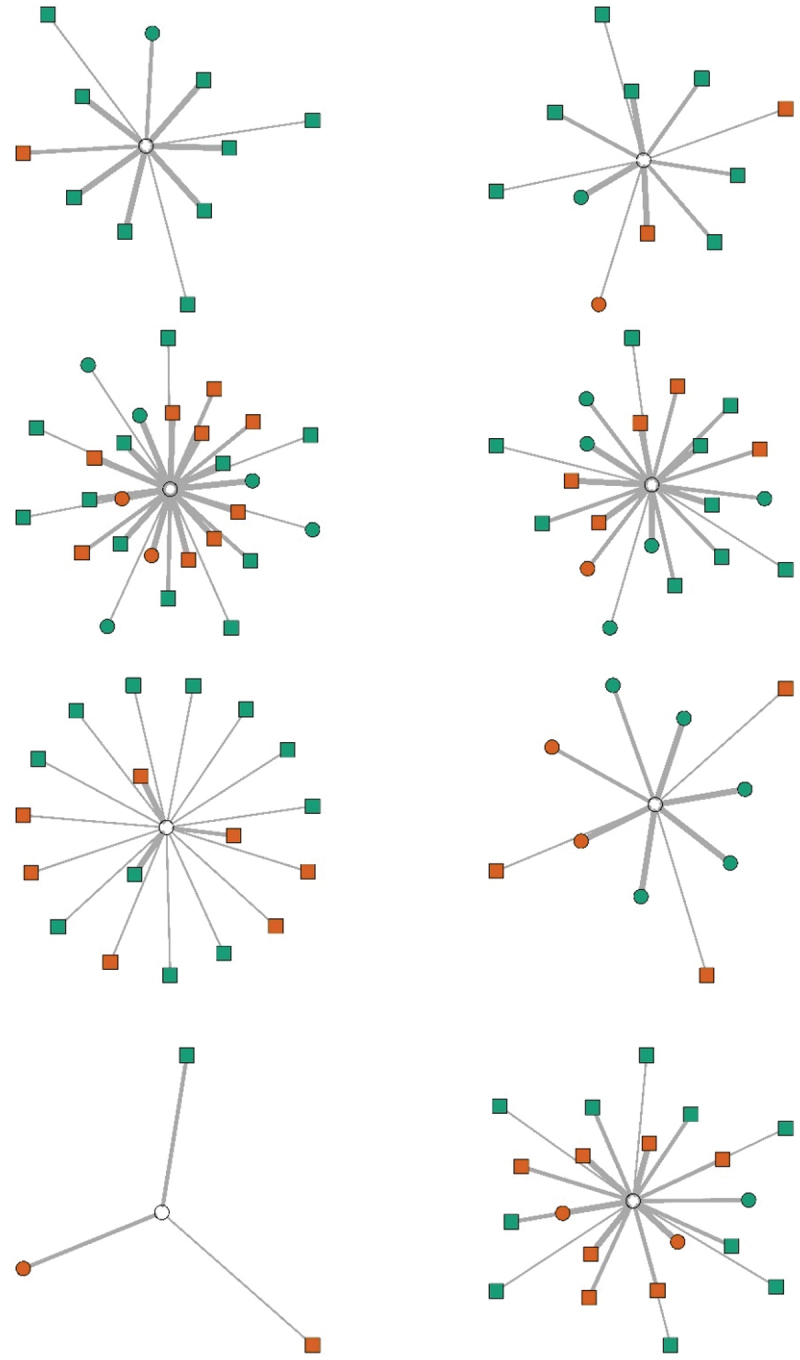
The same as 1a but for participants with 8 years or more since PhD.

### Research Time

Clinician-scientists with more research time, interacted on average more frequently with scientists than those with less research time, although the differences are small; (M_more research time_  = 2 vs M_less research time_ = 0, Table S2). The maximum number of scientists in the “frequent interactions” network of clinician-scientists with more research time was 6 compared to 1 for those with less research time. More time for research seemed to result in more extensive professional networks for knowledge exchange. For details, we refer to the Supplementary Material Table S2 and Figures S1a and S1b.

### Decisions to Mobilise Social Capital in Their Network

The qualitative thematic analysis revealed three decision-making processes of clinician-scientists in mobilising their social capital: (1) a deliberate decision to initiate connections, (2) a reactive approach, and (3) an ad-hoc approach. The first, most frequently mentioned, decision-making process was when clinician-scientists deliberately decided to approach others to exchange knowledge. The second was a reactive approach without much active decision making in advance: the clinician-scientists were approached by scientists and clinicians for questions about conducting research or the application of research findings, as a sparring partner or with a request for feedback.

I’m referring to … well, how medical practice deals with antibiotics and how we register things and that’s what we talked about. … Because they needed people to take part in that study. So he came to me … Yes, he’s looking for relevant practices. CS-10

A final decision-making process to mobilise social capital that we identified was when clinician-scientists decide ad-hoc on knowledge exchange. When clinician-scientists made use of opportunities for brief network interactions during moments initiated for different reasons.

We set up a date for us to see his house. And while we were visiting him, he told me about a study that they’re working on, and that they have all the data but haven’t written it up yet. CS-16

Our analysis indicated that the three ways of decision making might be interrelated, as illustrated by the quote below. A deliberate decision (to take the initiative) was made to foster a network in which, later on, mobilisation of resources could take place (or not) on the initiative of others (reactive) or decisions taken ad-hoc.

So I think if you invest in your relationships and just keep your ear to the ground and make sure you’re involved, then they know where to find you, and that makes it easier to pick up on things or sense what’s needed. CS-6

### Professional Development

In our quantitative results, we have shown how clinician-scientists are embedded in professional networks with clinician-scientists, scientists, and clinicians. As a result, they have access to and can mobilise different resources as part of their social capital. In analysing the qualitative data, we identified how this mobilisation what used for various goals where two broad categories emerged: some goals were related to the continuing professional development of others while other goals were related to their own growth. An example of a focus on the professional development of others, was identified when clinician-scientists mention how they strive to improve the ability of clinicians to use recent evidence from clinical research studies.

Precisely, I’m there and I can talk to them if need be … A general practitioner or a physician who says “I never do research”, well that’s unheard of, impossible. … Yes, because the foundation is knowledge, and knowledge is generated by science. And that sometimes changes over time and you build on that foundation and build something for individual patients, that’s obvious. That’s what evidence-based medicine is all about, narrowing it down to what it means for the individual patient. CS-8

In their connections with scientists, clinician-scientists mentioned that they felt responsible for ensuring that the research was relevant to clinical practice. Also, they contributed to the design of research studies so that clinicians’ participation in a study, for example, for data collection, would be feasible and not too burdensome. *I’m very good at talking to scientists about what is and isn’t feasible in practical terms. CS-14* Nevertheless, even though the clinician-scientists mentioned interactions with scientists, they did not speak in terms of contributing to the professional development of those scientists. In contrast, nearly twice as many identified fragments related to educating, teaching, or helping other clinicians.

In contrast to varying perceptions on contributing to the professional development of others, all clinician-scientists seemed aware of their activities contributing to their personal development. A large part of the described learning activities appeared to be carried out independently, without interaction with others. For example, they search for literature when faced with a clinical problem within the GP practice. Nevertheless, clinician-scientists also learn in interacting with others. They accentuated the necessity of a network to advance in their role.

I always used to think that you could do it all on your own but you can’t do it on your own, just like a clinician can’t. You can sit here by yourself but you won’t get anywhere that way. So you need other people, you need a network, you need your friends, you need partners – even as a scientist, you need partners. You need people who have your back. CS-5

### Relations Between Access, Mobilisation, and Professional Development

Clinician-scientists connect with others, mobilising social capital for their own professional development and that of others. Regardless of the overall size of their network or the frequency of interaction within their network, all clinician-scientists mentioned professional development of themselves and of clinicians as the two most clearly identified goals for using social capital. Those with less than eight years since PhD spoke more about their own professional development and less about the development of others. When clinician-scientists interact with fewer than the mean number in their networks, their own professional development was less prominent because of interactions with others and more prominent individually through reading the literature.The same was observed for clinician-scientists with little research time and those with more than eight years since PhD.

When clinician-scientists frequently, occasionally, or sporadically interacted with fewer than the mean number in their networks, mobilisation of their social network appeared to be focused on the professional development of others, especially clinicians, and not so much on their own knowledge development in interaction with others. Those with a more extensive network in one of the three frequency levels were often clinician-scientists with more than eight years since PhD and more research time. These respondents mostly talked about their own professional growth due to the interactions in their network or from being a clinician-scientist.

Clinician-scientists who, irrespective of the frequency of interaction, had interactions with more network connections described their approach in terms of being available for others (reactive approach) but talk less in terms of actively approaching others to learn or help develop professionally (deliberate decision to initiate contact). Respondents who primarily indicated that they take the initiative to exchange knowledge with others on scientific evidence for clinical practice, had a smaller network in all three levels of frequency. Experienced respondents apparently found a balance between being active and reactive. Less experienced clinician-scientists seemed to choose a reactive approach and ad-hoc decision-making more.

## Discussion

Clinician-scientists have a pivotal role in connecting science and clinical practice, [1,2] thus contributing to the continuing professional development of all those they connect with, including their own continuing professional development. The current study contributes to the literature by increasing our understanding of how the social capital of clinician-scientists adds to their continuing professional development and the continuing professional development of scientists and clinicians.

Based on the qualitative results, we observe three ways clinician-scientists use their social network for professional development. First, some clinician-scientists seem to use their network mainly one-directional, focusing on their own continuing professional development. Second, another group of clinician-scientists seem to seek bi-directional interactions with both scientists and clinical practitioners, thereby contributing to their own and the continuing professional development of others. Third, some clinician-scientists seem to mobilise their network only to a limited extent, prefer more individual learning activities (e.g. searching for literature).

Interestingly, based on the quantitative results for all clinician-scientists, the frequency of interactions indicates that clinician-scientists mobilise their resources with clinicians, scientists and primarily fellow clinician-scientists. This result suggests a homophily or similarity effect [17]. Homophily may be around different things but people with whom they have a common identity. However, earlier work showed that clinician-scientists do not all similarly perceive their social identity [27]. As such, it is questionable whether other clinician-scientists may be seen as similar and, as a result, interact more frequently. Alternatively, the knowledge exchange on scientific evidence for clinical practice may occur more frequently with other clinician-scientists due to other mechanisms; this asks for future research.

Generally, the quantitative results suggest that the less-experienced clinician-scientists seem to have, on average, a larger number of clinician-scientist with whom they frequently interact. For less-experienced clinician-scientists, interacting with clinician-scientists in their network might be helpful at the start of their careers and thus support their continuing professional development. In contrast, experienced clinician-scientists have more clinicians in the network with whom they interact sporadically. While cautiously interpreting these descriptive results, it is consistent with the literature that weak ties are essential for innovation for providing information and resources to help people (here clinicians) and supporting relations between groups within and between organisations [20,29]. Clinician-scientists with more research time, on average, interacted slightly more frequently with scientists than those with less research time. This would be consistent with the so-called bridging role of clinician-scientists between clinic and research [2,30].

The other qualitative findings indicate three decision-making processes in how clinician-scientists mobilise their social capital. Many clinician-scientists take a deliberate decision and initiative themselves to approach their network and thereby mobilise resources. They also behave reactively: after making sure that they are known in their network, they are available to answer questions or give feedback on ideas. The third approach is ad hoc when clinician-scientists do not connect with others with an explicit goal of knowledge exchange but take advantage of opportunities that arise during other (more informal) interactions. Future research is necessary to investigate these three distinct ways of decision-making. Based on the results of our study, the impression arises that clinician-scientists recognise the value of networking, as was expressed by the clinician-scientist that explicitly stated that others, colleagues, are needed to do your work. In the design of Continuing Professional Devlopment (CPD), formal training could provide learning opportunities in which clinician-scientists can develop their networking skills, specifically targeted at their bridging function between research and clinic. Furthermore, Continuing Medical Education (CME), aimed at clinicians, could create formal network opportunities between clinicians, scientists and clinician-scientists, for instance by organising events on topics relevant for research and clinic. Measurement of CME or CPD outcomes could pay attention to changes in the network composition, as this is an indication of clinician-scientists’ bridging role. Given the dynamic and complex role of clinician-scientists, creating a learning community for clinician-scientists could support them in their lifelong learning as clinician-scientist. Moreover, clinician-scientists see themselves as a bridge between research and practice when they express their reasons for mobilising their professional network, i.e. ensuring impact and using empirical evidence. Regarding differences between clinician-scientists with respect to the frequency of their interactions, it is important to note that numbers are small and the overall difference in the total frequency of interactions with scientists between the two groups was limited.

## Strengths and Limitations

This study is innovative since it is the first study that investigates a sample of clinician-scientists’ social networks and how the networks contribute to obtaining their individual goals (i.e. mobilising social capital). Dual-role professionals may sometimes be located within one single organisation, such as healthcare professionals who grow in parallel careers as managers or may exert their different roles in separate organisations [3]. Such dual-role professionals occur in the medical field [1,8] and other fields, such as education [31] and management [32]. As such, our study is also relevant to other professional domains. The limited number of participants does not afford an understanding of why some clinician-scientists possess the network composition that they do. Regarding differences between clinician-scientists with respect to the frequency of their interactions, it is important to note that numbers are small and the overall difference in the total frequency of interactions with scientists between the two groups was limited. Imaginable is that the context in which clinician-scientists work influences whether they are intentional in connecting and interacting with others. Social capital may not be mobilised because of unfavourable contextual factors [14]. Not many clinician-scientists in our domain exist, and hence, our sample was limited and thus does not allow to test for significant differences or prognostic factors. Since the descriptive results suggest differences in clinician-scientists’ professional networks, we recommend a follow-up study with more statistical power, which will have to be conducted in a setting in which clinician-scientists are more abundant. Also, we focussed on one-on-one interactions with other professionals. As a result, we did not capture all the connections because some respondents interact with groups (for example, in continuing education events). Furthermore, recall bias perhaps resulted in the generation of incomplete networks, although the participants were stimulated to add names to their network throughout the interview [33]. In future research, clinician-scientists could be followed for a longer period of time, tracking their real-time interactions with peers [34]. Nevertheless, there are no reasons why recall bias would be different between the groups. Finally, in our data collection, we specifically asked about their interactions with others on scientific knowledge. Consequently, unintentional, ad-hoc, and small-scale activities remained hidden. Collecting such data would ask for other methods such as prolonged observation studies. In future research, it would be valuable to broaden the scope to clinician-scientists in other healthcare areas. This would also increase the power of the study and go beyond the explorative character of the current study.

## Conclusion

This study provides insights into the professional networks of clinician-scientists in general practice and elderly care medicine. Less experienced clinician-scientists seem to connect mostly to similar others (i.e. clinician-scientists), whereas experienced clinician-scientists have interactions in a more diverse network but with a lower frequency. Having more research time seems to be beneficial for interactions with scientists. This provides clinician-scientist the opportunity to act as a so-called bridge between clinic and research. Clinician-scientists mobilise social capital in different ways to achieve goals – deliberate, reactive or ad-hoc. Our results suggest that the constellation of professional relationships (“with whom”) is equally vital as the knowledge exchange itself (“what”). This professional network will help to harvest the opportunities for knowledge exchange available in clinician-scientists’ professional networks. Clinician-scientists show different decision-making behaviours in interacting with clinicians, scientists and fellow clinician-scientists. Supporting interventions need to be aligned with that. Another practical implication of our work may be that facilitating interactions with fellow clinician-scientists, for example at CME or CPD events, could help novice clinician- scientists to become competent, immediately at the start of their career, of their important role in supporting the professional development of their colleages through bridging science and clinical practice.

## Supplementary Material

Supplemental Material

## Availability of Data and materials’ Statement

The datasets used and/or analysed during the current study are available from the corresponding author on reasonable request.

## Abbreviations

N: Number
CS: Clinician-scientist
M: Mean
CPD: Continuing Professional Development
CME: Continuing Medical Education
