# Supplementary material for: How Clinician-Scientists Access and Mobilise Social Capital and Thus Contribute to the Professional Development of Their Colleagues in Their Networks
Source: J CME. 2024 Nov 3;13(1):2421129. doi: 10.1080/28338073.2024.2421129 (PMC11536690; doi:10.1080/28338073.2024.2421129)
Supplement: Supplemental Material [file ZJEC_A_2421129_SM2106.docx]

# Appendices

**Interview guide**

| ***Question*** |
| --- |
| ***Name generator*** |
| “In the past year, with whom did you exchange knowledge on scientific evidence for clinical practice?”  *Please write their first names on a sticky note*  Follow-up questions for eliciting names until saturation is reached:  - In the past year, with whom *from clinical practice* did you discuss clinically relevant research?  - In the past year, with whom *from the field of research* did you discuss problems of clinical practice?  - Which of the people in your network to you consider friends (data on friendship relations was omitted from the manuscript overall) |
| From the people whom you just mentioned, whom of these are:   - clinicians? - scientists? - clinician-scientists? - if neither of the above, what is their role?   *(the interviewer added a C, S, CS to the sticky note)* |
| Please post the sticky-notes with the names of their professional connections on a map with three concentric circles, reflecting interactions that occurred frequently (the inner circle), occasionally (the second circle), or sporadically (the outer circle). |

Table S1: Experience, professional network size and network composition of the 15 clinician scientists. More experienced are those with equal to or more than 8 years research experience after their PhD; Less experienced are this with less than 8 years research experience after their PhD.

| **Frequency of interactions** | | **Mean** | **Min** | **Max** | **Mean** | **Min** | **Max** |
| --- | --- | --- | --- | --- | --- | --- | --- |
|  |  | **More experienced** | | | **Less experienced** | | |
| **Frequently** | |  |  |  |  |  |  |
| Clinicians | | 1 | 0 | 4 | 1 | 0 | 3 |
| Scientists | | 1 | 0 | 6 | 1 | 0 | 4 |
| Clinician-Scientists | | 1 | 0 | 8 | 3 | 2 | 4 |
| **Occasionally** | |  |  |  |  |  |  |
| Clinicians | | 1 | 0 | 3 | 2 | 0 | 4 |
| Scientists | | 1 | 0 | 3 | 1 | 0 | 3 |
| Clinician-Scientists | | 2 | 0 | 4 | 2 | 0 | 5 |
| **Sporadically** | |  |  |  |  |  |  |
| Clinicians | | 3 | 0 | 8 | 1 | 0 | 3 |
| Scientists | | 1 | 0 | 3 | 1 | 0 | 1 |
| Clinician-Scientists | | 1 | 0 | 5 | 1 | 0 | 3 |

Table S2: Research time, professional network size and network composition of the 15 clinician scientists. More research time means ≥40% of their workweek, and less research time means <40% of their workweek.

| **Frequency of interactions** | **Mean** | **Min** | **Max** | | **Mean** | **Min** | **Max** | |
| --- | --- | --- | --- | --- | --- | --- | --- | --- |
|  | **≥ 40%** | | | **< 40%** | | | |  |
| **Frequently** |  |  |  | |  |  |  | |
| Clinicians | 1 | 0 | 4 | | 2 | 0 | 4 | |
| Scientists | 2 | 0 | 6 | | 0 | 0 | 1 | |
| Clinician-Scientists | 3 | 0 | 8 | | 2 | 0 | 4 | |
| **Occasionally** |  |  |  | |  |  |  | |
| Clinicians | 1 | 0 | 4 | | 1 | 0 | 3 | |
| Scientists | 1 | 0 | 3 | | 2 | 0 | 3 | |
| Clinician-Scientists | 3 | 0 | 5 | | 1 | 0 | 3 | |
| **Sporadically** |  |  |  | |  |  |  | |
| Clinicians | 3 | 0 | 8 | | 1 | 0 | 3 | |
| Scientists | 1 | 0 | 3 | | 1 | 0 | 1 | |
| Clinician-Scientists | 1 | 0 | 5 | | 1 | 0 | 3 | |


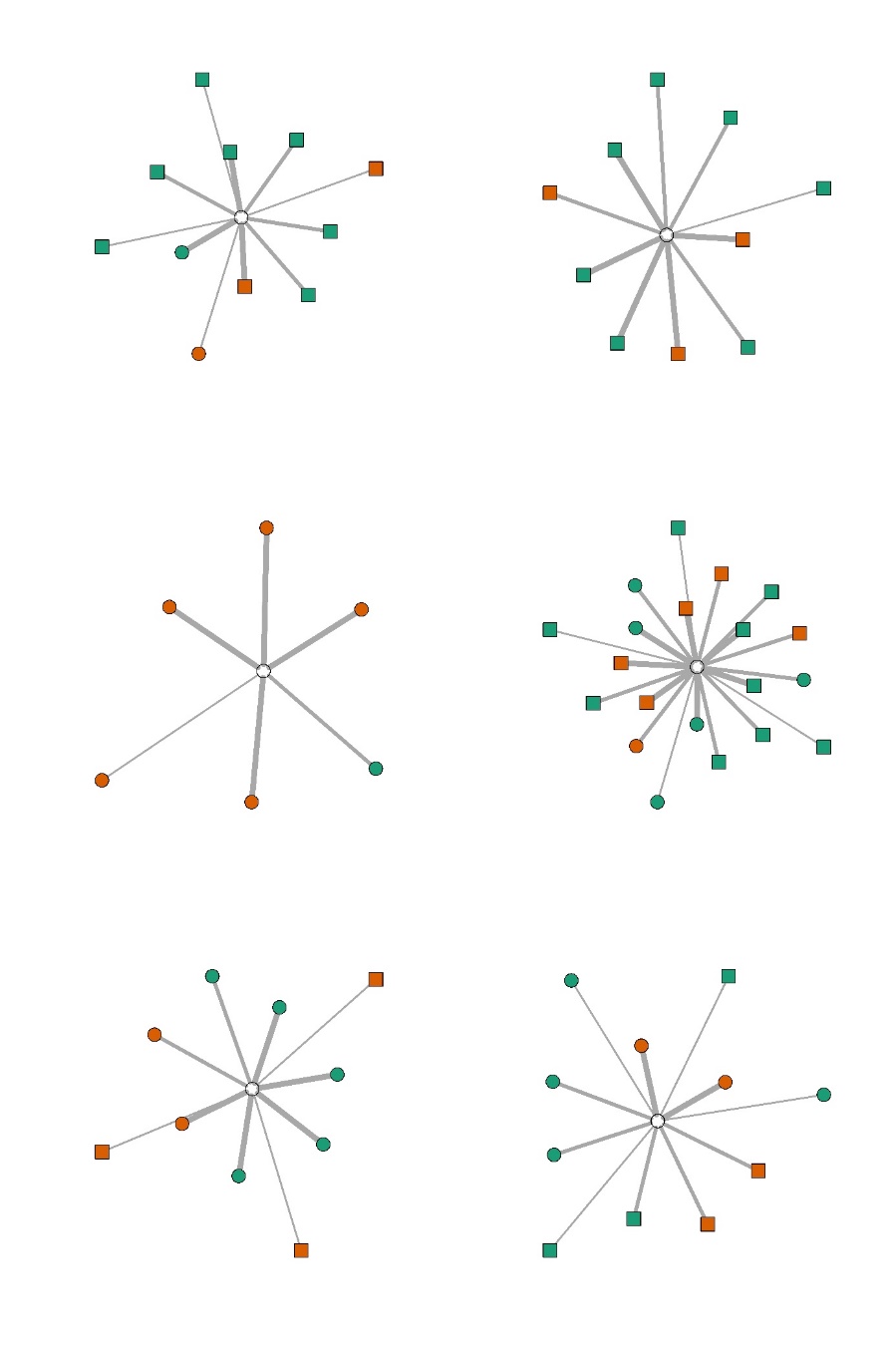
Figure S1a: Network visualisations for participants with < 40% of research time. The orange nodes are clinician-scientist which equals same profession while the green nodes are clinician or scientist. The nodes depicted as a circle ware considered friend, and square were not. The thick lines give an impression of frequent, the thin lines of occasional, and the very thin line of sporadical interactions.


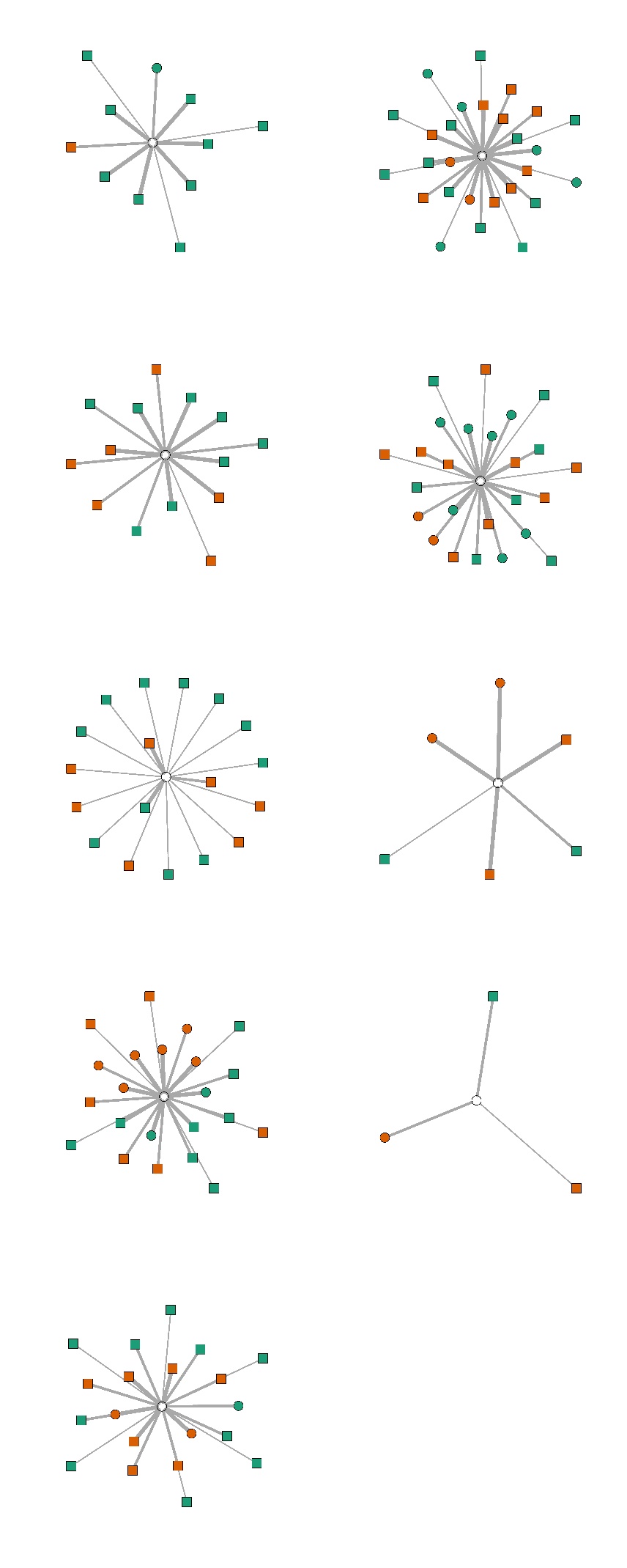
Figure S1b: The same as 2a but for participants with ≥ 40% research time.
